# Outer brain barriers in rat and human development

**DOI:** 10.3389/fnins.2015.00075

**Published:** 2015-03-16

**Authors:** Christian B. Brøchner, Camilla B. Holst, Kjeld Møllgård

**Affiliations:** ^1^Department of Cellular and Molecular Medicine, Faculty of Health and Medical Sciences, University of CopenhagenCopenhagen, Denmark; ^2^Department of Oncology, Copenhagen University HospitalCopenhagen, Denmark

**Keywords:** arachnoid blood-CSF barrier, development, outer CSF-brain barrier, pial microvessel blood-CSF barrier, SSEA-4, subarachnoid space, YKL-40

## Abstract

Complex barriers at the brain's surface, particularly in development, are poorly defined. In the adult, arachnoid blood-cerebrospinal fluid (CSF) barrier separates the fenestrated dural vessels from the CSF by means of a cell layer joined by tight junctions. Outer CSF-brain barrier provides diffusion restriction between brain and subarachnoid CSF through an initial radial glial end feet layer covered with a pial surface layer. To further characterize these interfaces we examined embryonic rat brains from E10 to P0 and forebrains from human embryos and fetuses (6–21st weeks post-conception) and adults using immunohistochemistry and confocal microscopy. Antibodies against claudin-11, BLBP, collagen 1, SSEA-4, MAP2, YKL-40, and its receptor IL-13Rα2 and EAAT1 were used to describe morphological characteristics and functional aspects of the outer brain barriers. Claudin-11 was a reliable marker of the arachnoid blood-CSF barrier. Collagen 1 delineated the subarachnoid space and stained pial surface layer. BLBP defined radial glial end feet layer and SSEA-4 and YKL-40 were present in both leptomeningeal cells and end feet layer, which transformed into glial limitans. IL-13Rα2 and EAAT1 were present in the end feet layer illustrating transporter/receptor presence in the outer CSF-brain barrier. MAP2 immunostaining in adult brain outlined the lower border of glia limitans; remnants of end feet were YKL-40 positive in some areas. We propose that outer brain barriers are composed of at least 3 interfaces: blood-CSF barrier across arachnoid barrier cell layer, blood-CSF barrier across pial microvessels, and outer CSF-brain barrier comprising glial end feet layer/pial surface layer.

## Introduction

For decades the meninges have been considered to be no more than a protective shield, but increasing evidence suggests that the complex outer barriers of the brain also function as morphogenic signaling centers (Siegenthaler and Pleasure, 2011; Decimo et al., 2012), dynamic transport systems (Yasuda et al., 2013), a stem cell niche (Decimo et al., 2012) and regulators of immune cell entry to the CNS (Stolp et al., 2013). Outer brain barriers have so far been treated as a single entity although it is well-established that they are composed of several independent cellular components. In this study we aimed to combine a short review of what is known in the field with new data describing arachnoid, pial, and brain surface development in rat and human.

### The meninges

In addition to the blood-brain and the classical blood-CSF (across the choroid plexus) barriers, the arachnoid barrier is a third barrier forming an important interface between blood and CSF in the brain. Dyes injected into the blood tend to stain dura but not the arachnoid suggesting that the arachnoid rather than the dura prevents entry of blood-borne substances into the subarachnoid space (Broman, 1949). Later, ultrastructural electron microscopical (EM) and freeze fracture studies described the arachnoid as a multi-layered epithelium with tight junctions between cells of the outer continuous 1–3 cellular layers—the arachnoid barrier cell layer—that forms an effective seal covering the inner dural surface (Nabeshima et al., 1975; Rascher and Wolburg, 1997). The pia and the inner layer of arachnoid comprise one cell type—the leptomeningeal cell—covering the outer-most layer of nervous tissue of the brain, the glia limitans, which is composed of a dense multilayered meshwork of astrocytic processes covered by an outer basement membrane. The layers of developing human meninges were inve*s*tigated and defined by O'Rahilly and Muller (1986) starting with the primary meninx comprising neural crest-derived cells (Decimo et al., 2012) that initially surround the neural tube and at around 6th week post-conception (wpc) are present around most parts of the brain. Later 6 layers develop consisting of subcutaneous tissue, the skeletogenous layer (the dense mesenchyme between the subcutaneous layer and at first the primary meninx), dura mater, the dural limiting layer (probably contributing to both dura and arachnoid), leptomeninx (arachnoid and pia), and cerebral wall (O'Rahilly and Muller, 1986).

Differentiation of the meninges also involves formation of the subarachnoid space. Thus, the spaces that appear in the inner reticulated layer of the primary meninx become confluent and develop into the subarachnoid space and cisterns during late embryonic and early fetal period. Leptomeningeal cells condense at the inner surface of the dura and become arachnoid barrier cells and at the brain surface they form the pial surface layer. In newborn rat and in early fetal human brain the subarachnoid space extends from the arachnoid barrier cell layer to the basement membrane of the pial surface layer and contains CSF and blood vessels bridged by leptomeningeal cells that also form traversing arachnoid trabeculae (see Discussion).

Recent investigations have changed the established view of the meninges as merely protective membranes suggesting that they contribute to neural tissue homeostasis by secreting several trophic factors including FGF2, insulin-like growth factor-II and CXCL12/SDF-1 (Decimo et al., 2012) and they are highly responsive to principal mitogens and necessary for survival and growth of the developing brain (Decimo et al., 2012). In the fetal brain the meninges act as a morphogenetic center via secretion of retinoic acid which regulates neural migration and positioning, and organize the pial basement membrane, a critical anchor point for the radially oriented fibers of neuroepithelial stem cells and radial glial cells, through a variety of other secreted diffusible factors (Siegenthaler and Pleasure, 2011; Decimo et al., 2012).

### The end feet layer

The neuroepithelial cell layer of the prosencephalon and early telencephalon covered with basement membrane faces the outside world prior to a forth-coming subarachnoid space. Along with the differentiation of neuroepithelial cells to radial glia the radial glial end feet layer covered with a basement membrane and flattened leptomeningeal cells forms a continuous interface between the telencephalic wall and the early subarachnoid space. Glial transformation from radial glial cells to astrocytes begins in the second trimester in humans and between 25 and 28 wpc the subpial radial glial end feet layer seems to start transforming into a subpial glia limitans (Kadhim et al., 1988) consisting of interdigitating astrocytic processes and a few fibrous astrocytes. Kadhim et al. (1988) found that late subpial end feet were almost free of autophagic and lysosomal hyperactivity indicating that the end feet layer and subsequent glia limitans are created from radial glial cells that do not undergo lysosomal autolysis.

The morphology of the barrier interface over the surface of the brain is most complex during its early development. Thus, in addition to the adult barrier of tight junctions linking subdural arachnoid cells and endothelial cells of blood vessels in the subarachnoid space (Nabeshima et al., 1975), there is a whole array of specialized intercellular junctions over the pial surface of the brain. This has been described in the rat embryo where a progressive appearance of distinct junctional structures between the glial end feet was observed from E14; analysis of albumin distribution at the EM level suggested that the junctions may contribute to restriction of diffusion between the subarachnoid space and the brain extracellular fluid (Balslev et al., 1997). Nabeshima et al. (1975) noted gap junctions between end feet in the glia limitans in different mammals, which has been confirmed by others (e.g., Balslev et al., 1997; Feig and Haberly, 2011). The glia limitans has also been found to harbor specialized transmembrane protein complexes, and may contribute to neural regulation of blood flow through pial arteries, homeostatic regulation of K+ and water and modulation of neural activity (Feig and Haberly, 2011). It must therefore be perceived as an important interface in the brain barrier system, albeit studies regarding the barrier properties of this outer CSF-brain barrier are scarce.

### Brain barriers and terminology

Many names have been applied to the outer brain barriers, which complicate interpretation of results. The outer CSF-arachnoid barrier layer is identical with the blood-arachnoid barrier and the arachnoid barrier cell layer. The subarachnoid space with its contents extends from the arachnoid barrier cell layer to the outer CSF-brain barrier. The outer CSF-brain barrier changes during development from a radial glial end feet layer covered by the inner-most part of the pial surface layer which initially consists of a pial basement membrane and a condensed layer of single leptomeningeal cells to a glia limitans formed by astrocytic end feet and surface associated astrocytes covered by a pial basement membrane and scattered associated leptomeningeal cells. This outer CSF-brain barrier and its individual components have also been referred to as glial limiting membrane, external pial limiting membrane, glial end feet barrier, pia-arachnoid end feet barrier and other names, which sometimes interchange and therefore further obscure results. The arachnoid barrier cell layer and outer CSF-brain interface have also been classified as one barrier and labeled the pia-arachnoid-brain barrier and in some cases the outer CSF-brain barrier is completely ignored.

The prevailing classification of brain barriers in the developing brain has until now included an arachnoid barrier, a blood-brain barrier, a blood-CSF barrier (across the choroid plexus) and a CSF-brain barrier (across the ventricular zone) (Saunders et al., 2008, 2013). In this study we define brain interfaces that provide diffusion restriction as barriers, and taking into account the confusion in terminology, we define the terms used: The blood-CSF barrier, often only defined as the barrier across the choroid plexus, also encompass the barrier across the arachnoid barrier cell layer and the pial microvessels, since these interfaces provide a barrier between blood and the CSF. We name them the arachnoid blood-CSF barrier across the arachnoid barrier cell layer and the pial microvessel blood-CSF barrier across pial microvessels, with the proviso that the subarachnoid space in the very early embryonic brain does not exist yet nor does it contain CSF, since the choroid plexuses have not started their production yet. The barrier in the pial microvessels has been used as a surrogate for the blood-brain barrier (Allt and Lawrenson, 1997). This is incorrect since the neurovascular unit, which contributes to the fully differentiated blood-brain barrier (Engelhardt and Coisne, 2011) is not present in the subarachnoid space, and given the fact that pial microvessels are not part of the blood-brain interface, only the barrier across brain parenchymal blood vessels should be referred to as a blood-brain barrier proper (BBB). The CSF-brain barrier consists of both an outer CSF-brain barrier (described here) across the radial glial end feet layer covered by a pial surface layer and later in development glia limitans with accompanying pial basement membrane, and an inner CSF-brain barrier (generally referred to as CSF-brain barrier) across the ventricular zone cell layer.

### Outer brain barriers in rat and human development

The interfaces at the surface of the developing brain comprise the arachnoid blood-CSF barrier and outer CSF-brain barrier separated by a subarachnoid space containing pial microvessels, which form the pial microvessel blood-CSF barrier. As stated above, little is known about the changes in outer barriers of the brain during development. In addition the outer CSF-brain barrier has been largely ignored partly because of known lack of obvious tight junctions (Balslev et al., 1997). It is now evident that brain barriers are more than just the presence of tight junctions, and it is therefore necessary to characterize, divide and define these complex barriers at the surface of the developing brain. To this end, we examined embryonic rat brains from E10 to P0 and the outer brain barrier site in human forebrain samples from human embryos and fetuses (6–21st weeks post-conception) and adult brain samples using immunohistochemistry and confocal microscopy. An array of various specific antibodies was used against tight junctions (claudins), radial glial cells, glial cells, and neurons (BLBP, EAAT1, GFAP, MAP2).

In a recent study we found YKL-40 mRNA expression in meninges, telencephalon and choroid plexus and YKL-40 immunostaining of sites at all interfaces of the barriers in the developing human brain (Bjørnbak et al., 2014). Our results suggested that YKL-40 secretion from choroid plexus epithelium, leptomeningeal cells, and pericytes may play a main role associated with transport across the entire mesenchymal/neuroectodermal interface (Bjørnbak et al., 2014). Furthermore, the overall distribution of YKL-40 in the developing human forebrain was noted to be very similar to that of SSEA-4 in forebrains of embryos and early fetuses (Barraud et al., 2007) from this same collection. Therefore, we included antibodies against SSEA-4, YKL-40 and IL-13Rα2, an YKL-40 receptor.

## Materials and methods

### Tissue samples

Forebrains from two human embryos [7 and 31 mm crown-rump length (CRL)] and 11 fetuses (38–200 mm CRL) corresponding to 6–21st weeks post-conception (wpc) and two adult brain specimens were examined. The embryos and the fetuses were obtained from legal abortions, as previously described (Bjørnbak et al., 2014). The adult brain samples were donated for research following neurosurgery. Informed consent was obtained from all contributing women following oral and written information, in accordance with the Helsinki declaration II, and approved by the Research Ethics Committee of the Capital Region (KF–V.100.1735/90). Post-operational treatment consisted of immediate dissection of the samples into blocks, that were fixed for 12–24 h at 4°C in either 10% neutral buffered formalin, 4% Formol-Calcium, Lillie's or Bouin's fixatives. Rat embryos, 4 for each age at E12, 14, 16, 18, and P0 (the day of birth) fixed by immersion in either 10% neutral buffered formalin or Bouin's fixatives were available from a previous investigation (Balslev et al., 1997). Prior to paraffin embedding, the specimens were dehydrated with graded alcohols, and cleared in xylene. For single and double immunohistochemical experiments, 3–10 μm thick serial sections were cut in transverse, sagittal or horizontal planes, and placed on silanized glass slides.

### Immunohistochemistry

Sections were deparaffinized in xylene and rehydrated using graded alcohols following standard protocols. Endogenous peroxidase was quenched using a 0.5% solution of hydrogen peroxide in methanol for 15 min. Following rinses with TRIS buffered saline (TBS, 5 mM Tris-HCl, 146 mM NaCl, pH 7.6), non-specific binding was inhibited by incubation for 30 min with blocking buffer (Dako REAL™, S2022, Dako, Glostrup, Denmark) or 0.2% casein (C-7078, Sigma-Aldrich, St. Louis, MO, USA) at room temperature. Prior to overnight incubation at 4°C with primary antibodies diluted in blocking buffer, some antibodies required heat induced epitope retrieval with either citrate buffer (pH6) or TEG buffer (pH9) (Table 1). Before developing the signal, sections were washed with TBS. For bright field light microscopy analysis, the REAL™ EnVision™ Detection System, Peroxidase/DAB+ rabbit/mouse, (K5007, Dako, Glostrup, Denmark) was used for detecting mouse and rabbit primary antibodies. The sections were washed with TBS, followed by incubation for 10 min with 3,3′-diamino-benzidine chromogen solution. Positive staining was recognized as a brown color. The sections were counterstained with Mayers hematoxylin and dehydrated in graded alcohols followed by xylene and coverslipped with DPX mounting media. For double labeling immunofluorescence, sections were incubated for 30 min at room temperature with labeled polymer–HRP anti-mouse (EnVision™+ System/HRP, K4007, Dako, Glostrup, Denmark). In order to amplify the immunoreaction, this was followed by incubation with a tyramide signal amplification kit with Alexa Fluor 488-labeled tyramide (1:200, T20912, Life Technologies, Carlsbad, CA, USA) for 7 min at room temperature. Subsequently, the sections were incubated for 30 min at room temperature with biotin-SP-conjugated F(ab′)_2_ fragment donkey anti-rabbit antibodies (1:200, 711-066-152, Jackson ImmunoResearch Europe, Suffolk, United Kingdom) followed by streptavidin-conjugated DyLight 594 (1:200, SA5594, Vector Laboratories Europe, Peterborough, United Kingdom). Finally, a nuclear counterstain with DAPI (1:1000, D1306, Life Technologies, Carlsbad, CA, USA) was added for 3 min, before sections were coverslipped. Double labeling was performed with the combination of antibodies against BLBP and SSEA-4, YKL-40 and SSEA-4, YKL-40 and IL13-Rα2, and YKL-40 and EAAT1. Details of the primary antibodies including dilutions and suppliers are listed in Table 1. Staining specificity of the monoclonal YKL-40 antibody was thoroughly tested on the same material in a recent study (see Figure 1 in Bjørnbak et al., 2014), and in addition the polyclonal anti-YKL-40 used here resulted in an immunostaining identical to that of monoclonal YKL-40. Control sections were incubated with mouse IgG1, IgG2a or irrelevant rabbit antibodies, as well as subjected to omission of primary or secondary antibodies. These were always blank. The staining patterns were similar irrespective of different methods of fixation used as also emphasized in many studies based on the same collection (e.g., Møllgård and Jacobsen, 1984; Johansen et al., 2007).

**Table 1:** **List of primary antibodies**.

**Primary antibodies**	**Host IgG**	**Dilution**	**HIER**	**Producer**	**Code number**
BLBP	Rabbit IgG	1:2000	-	Millipore	ABN14
Claudin-11/OSP	Rabbit IgG	1:1500	-	Abcam	ab53041
Collagen 1	Rabbit IgG	1:500	citrate, pH6	Abcam	ab292
EAAT1	Rabbit IgG	1:400	TEG, pH9	Abcam	ab416
GFAP	Rabbit IgG	1:10000	TEG, pH9	Dako	Z0334
IL-13Rα2	Rabbit IgG	1:100	-	Proteintech	11059-1-AP
MAP2	Clon HM-2	1:10000	TEG, pH9	Sigma Aldrich	M4403
SSEA-4	Mouse IgG3	1:50/1:100	-	Millipore	MAB4304
YKL-40	Rabbit IgG	1:10	-	Proteintech	12036-1-AP
YKL-40	Mouse, IgG2kb	1:50/1:100	-	^*^	201.F9

HIER, Heat Induced Epitope Retrival. TEG, TRIS + ethylene glycol tetraacetic acid buffer. Producers, Abcam, Cambridge, United Kingdom; Millipore, Merck Life Science, Hellerup, Denmark. Dako, Glostrup, Denmark. Sigma-Aldrich, St. Louis, MO, USA. Proteintech, Manchester, United Kingdom.

* *Kind gift from Prof. Paul A. Price, UCSD, USA*.

For laser scanning confocal microscopy a Carl Zeiss LSM 780 was used. During image acquisition, a sequential scan procedure through the z-axis of the double-labeled sections was applied when applicable, covering in total 9–11 μm in depth. Confocal images were taken and analyzed throughout the z-axis of the section, and individual optical sections were stored as TIFF files using Zeiss ZEN Vision v10. Representative images were chosen for figure editing using Adobe Photoshop CS6.

## Results

The meninges and outer brain barriers form during embryogenesis and early fetal life. Many factors are involved in this dynamic process and are necessary for adequate brain development. O'Rahilly and Muller (1986) described development of the human meninges according to Carnegie stages, but have recently revised this staging model (O'Rahilly and Muller, 2010). In Table 2 a summary of important events in the development of the human meninges and outer CSF-brain barrier approximated to weeks post-conception (wpc) is provided. Results in Carnegie stages have been translated according to the revision by O'Rahilly and Muller (2010) and from mammals to human according to the work of Clancy et al. (2001).

**Table 2:** **Features of outer brain barrier and meningeal development**.

**WPC**	**Feature**	**Species**	**References**
6th	Appearance of a primary meninx around most parts of the brain	Human	O'Rahilly and Muller, 1986
	Amoeboid microglial cells penetrate the brain by crossing the pial basement membrane	Human	Verney et al., 2010
7th	The skeletogenous layer becomes visible	Human	O'Rahilly and Muller, 1986
	Differentiation of a leptomeningeal meshwork that is presumed to contain liquid and represent the future SAS	Human	O'Rahilly and Muller, 1986
8th	The dural limiting layer is almost complete in hindbrain and midbrain but only present in the area adjacent to the lateral fossa in the forebrain	Human	O'Rahilly and Muller, 1986
	The fenestrated sinusoids of the pia-arachnoid become non-fenestrated (E14)	Rat (E14)	Balslev et al., 1997
	Most of the cisternae of the adult is already present	Human	O'Rahilly and Muller, 1986
7–10th	Initial communication between the ventricular and subarachnoid compartments	Human and rat (E17)	Brocklehurst, 1969; Johansson et al., 2008
11th	Completion of the subpial end feet layer (E16) and claudin-11 positive arachnoid blood-CSF barrier (E18) and thereby appearance of a clearly defined subarachnoid space	Rat (E16 and E18)	Balslev et al., 1997
12–13th	Second wave of microglial cells penetrate the brain via the BBB and inner CSF-brain barrier	Human	Verney et al., 2010
12 (13–15th)	RGCs begin to transform into astrocytes	Rhesus monkey (E64) and newborn ferret	Schmechel and Rakic, 1979; Voigt, 1989
25–28th	Transition from subpial end feet layer to glia limitans	Human	Kadhim et al., 1988

*Data were mainly based on human material and approximated to weeks post-conception. It is clearly stated when results from other mammals are used. The work of Clancy et al. (2001) was used for conversion to estimated human age*.

We aimed to characterize the interfaces at the surface of the developing brain revealing a claudin-11 positive arachnoid blood-CSF barrier, collagen 1 positive subarachnoid space and pial surface layer, SSEA-4/YKL-40 positive leptomeningeal cells and SSEA-4/YKL-40/IL-13Rα2/BLBP positive radial glial end feet. Each interface is described and discussed in the context of possible functional significance for the developing brain.

### The arachnoid blood-CSF barrier becomes increasingly claudin-11 positive during rat and human brain development

Previously we have described the developing rat glial end feet barrier prior to the appearance of the arachnoid membrane and thus before the establishment of a well-defined subarachnoid space (Balslev et al., 1997). In a recent study of the embryonic mouse brain immunostained for claudin-11, a distinct reactivity corresponding to the developing arachnoid blood-CSF barrier was found (Whish et al., 2015). Therefore, we decided to test claudin-11 as a possible marker for the arachnoid blood-CSF barrier development in rat and human in order to compare this barrier with the developing outer CSF-brain barrier described previously in the rat (Balslev et al., 1997).

**Figure 1:**
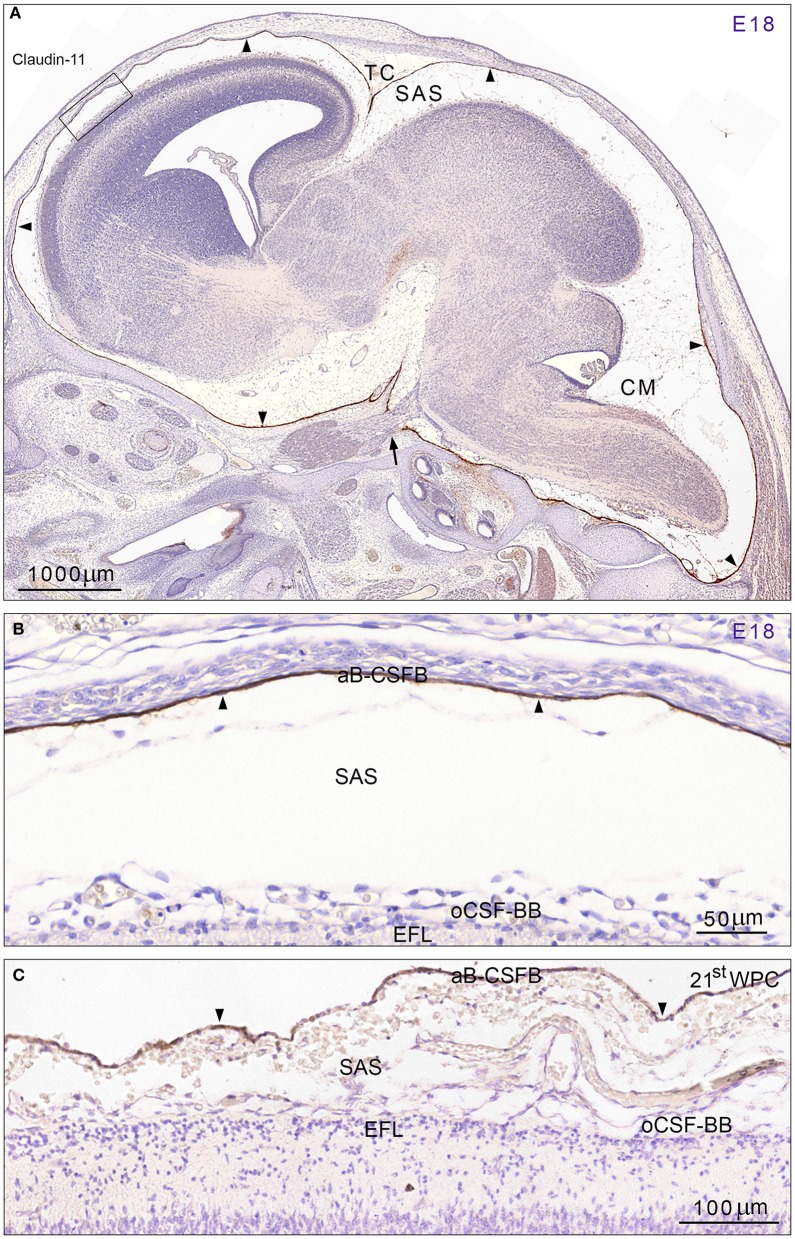
**Distribution of claudin-11 immunoreactivity in sagittal sections of E18 rat (A,B) and 21st wpc human (C) brain. (A)** Immunostaining for claudin-11 in a developing rat brain at E18 demonstrates a strong reactivity of the entire arachnoid barrier cell layer (= arachnoid blood-CSF barrier (aB-CSFB)) (*arrowheads*) in marked contrast to unstained leptomeningeal cells in the subarachnoid space (SAS) and in the entire brain. Cisterns at the base of the brain and cisterna magna (CM) are well-developed. Note the very strongly stained arachnoid covering of the tentorium cerebelli (TC) and the interrupted barrier cell layer where the trigeminal nerve perforates the dura-arachnoid (*arrow*). The area outlined with a *rectangle* is shown at higher magnification in **(B)** and demonstrates that the barrier cell layer (aB-CSFB) covering the forebrain consists of a strongly stained single cell layer. **(C)** At mid-gestation the human fetal arachnoid barrier cell layer (aB-CSFB) is also formed by a single claudin-11 positive cell layer. In **(B,C)** note that neither the E18 rat radial glial end feet layer (EFL) and the pial surface layer forming the outer CSF brain barrier (oCSF-BB) facing the subarachnoid space (SAS) nor the human fetal radial glial end feet layer (EFL) and pial surface layer show claudin-11 immunoreactivity. *Scale bars*: **(A)** 1000 μm, **(B)** 50 μm, **(C)** 100 μm.

Early developing rat brain from E10 to E14 showed a complete lack of claudin-11 immunoreactivity. At E15 a faint immunostaining for claudin-11 was present corresponding to loosely packed arachnoid barrier cells at the base of the brain and to the developing barrier layer of the future tentorium cerebelli (not illustrated). At E18 meninges, the subarachnoid space and cisterns were fully developed and immunostaining for claudin-11 demonstrated a strong distinct reactivity of the entire arachnoid blood-CSF barrier (Figures 1A,B). The barrier cell layer was particularly prominent corresponding to tentorium cerebelli and the base of the brain and interrupted only where cranial nerves penetrated the combined dura-arachnoid (Figure 1A). The arachnoid barrier cell layer of the forebrain consisted of a single cell layer shown in Figure 1B. In late embryonic human brain claudin-11 reactivity was first observed as a loose faintly stained meshwork of leptomeningeal cells surrounding the lower brain stem. At 8th wpc arachnoid barrier cells at tentorium cerebelli and at the base of the brain were positive. In the second trimester meninges, the subarachnoid space and cisterns were fully developed. At mid-gestation the arachnoid blood-CSF barrier of the forebrain consisted of a single claudin-11 positive cell layer (Figure 1C).

### The subarachnoid space is defined by collagen 1 immunoreactivity

**Figure 2:**
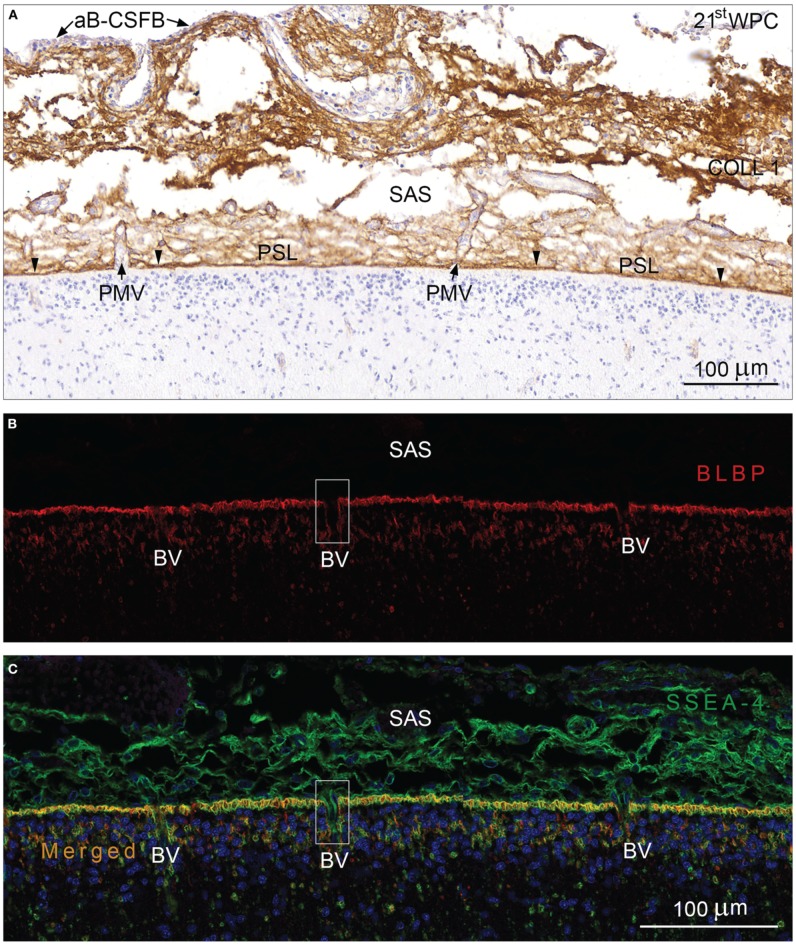
**Arachnoid blood-CSF barrier, subarachnoid space, pial surface, and glial end feet layers immunostained for collagen 1 (A) and double immunolabeled with antibodies against BLBP and SSEA-4 (B,C) from occipital cortex of a 21st wpc human fetus. (A)** Immunostaining for collagen 1 depicts the limitation of the subarachnoid space (SAS) of a 21st wpc human fetus. The arachnoid barrier cell layer (aB-CSFB) is unstained (*arrows*) and the strongly immunostained pial surface layer (PSL) faces an unstained glial end feet layer. The basement membranes of the pial microvessels (BV) loose their stainability abruptly when the vessels enter the end feet layer and fuse with the inner-most part of the PSL (*arrowheads*). **(B)** In a section adjacent to that shown in **(A)**, the entire end feet layer is immunolabeled with anti-BLBP corresponding to the radial glial end feet. Note the spaces in the end feet layer where blood vessels penetrate into the parenchyma. **(C)** Double-labeling with antibodies against BLBP and SSEA-4, nuclear stained with DAPI, reveals an evenly distributed co-localization in the end feet layer. Where pial microvessels penetrate into the marginal zone, the perivascular canals are lined with BLBP, revealing Virchow-Robins space (illustrated with rectangles in **B,C**). The subarachnoid space contains SSEA-4 positive leptomeningeal cells but shows no BLBP reactivity. **(B,C)** same magnification. *Scale bars*: 100 μm.

**Figure 3:**
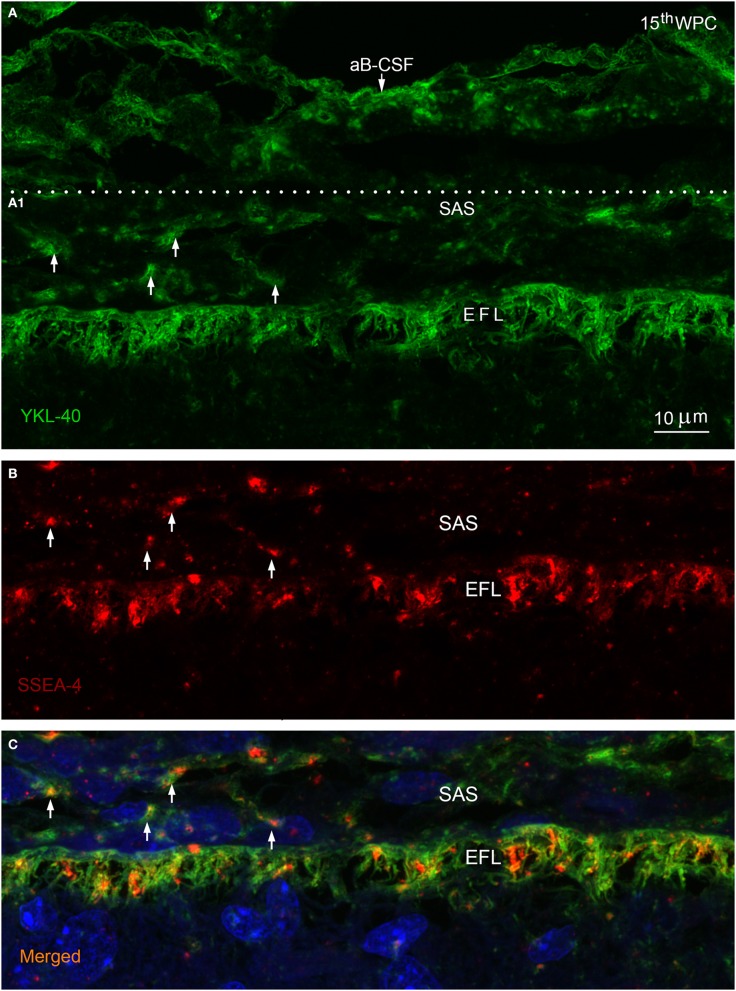
**Arachnoid blood-CSF barrier, subarachnoid space, pial surface, and glial end feet layers double immunolabeled with antibodies against YKL-40 and SSEA-4 (A–C) in parietal cortex of a 15th wpc human fetus**. Parietal cortex of a 15th wpc fetus, immunolabeled with anti-YKL-40 **(A)** and anti-SSEA-4 **(B)**, counterstained with DAPI and merged **(A1,B)** in **(C)**. The images are representations of a stacked series of 10 steps, with 1 μm intervals, processed as maximum projection intensity. The staining of the end feet layer (*EFL*) shows multiple delicate and curled end feet membranes. YKL-40 immunoreactivity is also present in the arachnoid blood-CSF barrier (aB-CSFB in **(A)** and in leptomeningeal cells in the subarachnoid space (*arrows* in **A1**). In **(B)** the pattern of SSEA-4 distribution is seemingly equal to that of YKL-40, and the merged image of the stacked images confirms the co-localization of YKL-40 and SSEA-4 in leptomeningeal cells within the subarachnoid space (SAS) and in the end feet layer of the radial glial cells **(C)**. *Scale bar*: 10 μm.

The second trimester human fetal subarachnoid space between the arachnoid blood-CSF barrier and the outer CSF-brain barrier contained numerous bundles of collagen, many blood vessels and a meshwork of leptomeningeal cells forming trabeculae. Immunostaining for collagen 1 clearly delineated the subarachnoid space as shown in a section from a mid-gestational human fetus in Figure 2A. The arachnoid barrier cell layer was unstained in contrast to the strongly immunostained pial surface layer which faced an unstained glial end feet layer. The basement membranes of the pial blood vessels lost their collagen 1 immunoreactivity upon entering the end feet layer, revealed as a continuous layer of glial end feet only interrupted by penetrating blood vessels after immunostaining for brain specific lipid-binding protein (BLBP) (Figure 2B). The leptomeningeal cells in the subarachnoid space including the arachnoid barrier cell layer were positively stained for both YKL-40 and SSEA-4 (Figure 3) but not for BLBP (Figure 2B). Where blood vessels penetrated the end feet layer the subarachnoid space extended down into a subpial compartment in the marginal zone. Double-labeling with antibodies against BLBP and SSEA-4, showed SSEA-4 immunoreactivity of the pericytes and BLBP defined the outer invaginating surface of the end feet layer, corresponding to the perivascular Virchow-Robin space between vascular basement membrane and surface of end feet (Figures 2B,C). The Virchow-Robin space is obliterated when the glial and endothelial basement membranes join (Brinker et al., 2014) consistent with depletion of BLBP and SSEA-4 immunoreactivity.

### From radial glial end feet layer, characterized by BLBP-, SSEA-4 and YKL-40 immunoreactivity, to glia limitans

#### Radial glial end feet layer in fetal human brain

In late embryos and early fetuses immunostained for BLBP, SSEA-4, and YKL-40 the entire parenchymal lining of the outer CSF-brain barrier, corresponding to radial glial end feet, exhibited strong membranous immunoreactivity of the conically or deltoid shaped end feet with simple straight membranes. The parenchymal lining in the second trimester showed multiple curly closely apposed delicate end feet membranes best appreciated in a stacked series of confocal images of YKL-40 stained marginal zone (Figure 3A). At mid-gestation the individual end feet appeared as narrow densely packed parallel tubules outlined by rather straight apical and apical-lateral cell membranes (not shown). Double immunolabeling with antibodies against BLBP and SSEA-4 (Figure 2C) and with antibodies against YKL-40 and SSEA-4 (Figures 3A–C) demonstrated that all end feet possessed a considerable overlapping membrane-associated immunoreactivity for BLBP, SSEA-4 and YKL-40.

#### Receptors and transporters associated with the end feet layer

**Figure 4:**
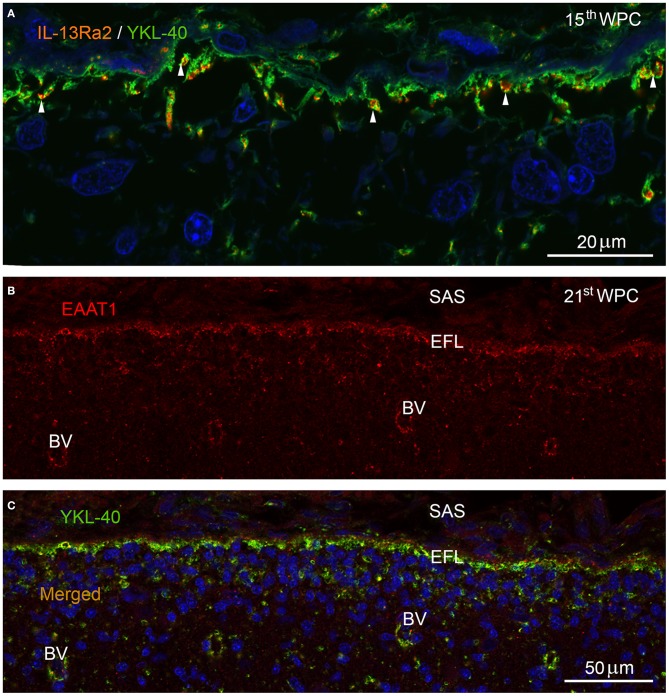
**Marginal zone in parietal cortex of a 15th wpc human fetus double immunolabeled with antibodies against YKL-40 and IL-13Rα2 (A) and in occipital cortex of a 21st wpc human fetus (B,C) labeled with antibodies against EAAT1 (B) and against EAAT1 and YKL-40 (C)**. Parietal cortex of a 15th wpc fetus, immunolabeled with antibodies against IL-13Rα2 and YKL-40, merged and nuclear counterstained with DAPI **(A)**. The YKL-40 immunoreactive end feet layer contains evenly distributed interleukin-13Rα2 receptors (white arrowheads). **(B)** The excitatory amino acid transporter (EAAT1) is visualized in occipital cortex of a 21st wpc fetus, below the subarachnoid space (SAS) within the end feet layer (EFL) and in surrounding parenchymal blood vessels (BV). The merged image with YKL-40 **(C)** shows a similar distribution within the end feet layer and around the blood vessels. **(A)** *Scale bar:* 20 μm. **(B,C)** same magnification. *Scale bar:* 50 μm.

IL-13Rα2, which was recently shown to function as an YKL-40/CHI3L1 receptor (He et al., 2013) and EAAT1/GLAST, a well-known glutamate-aspartate influx transporter (also known as *Slc1a3*), were used as representative examples to illustrate dynamic functions of the end feet layer. Double-immunolabeling of brains from human fetuses in the second trimester with antibodies against IL-13Rα2 and YKL-40 (Figure 4A) showed that the entire continuously YKL-40 immunoreactive end feet layer contained evenly distributed IL-13Rα2 positive membrane domains in infoldings of the end feet surface membrane. Immunostaining with antibodies against the excitatory amino acid influx transporter EAAT1 resulted in immunopositive punctate or reticular structures along the membranes of the end feet layer and surrounding blood vessels in the outer neocortex at mid-gestation (Figure 4B). Double-immunolabeling with antibodies against EAAT1 and YKL-40 (Figure 4C) showed a similar distribution of influx transporter and YKL-40 within the end feet layer and around blood vessels.

#### Glia limitans in adult human brain

**Figure 5:**
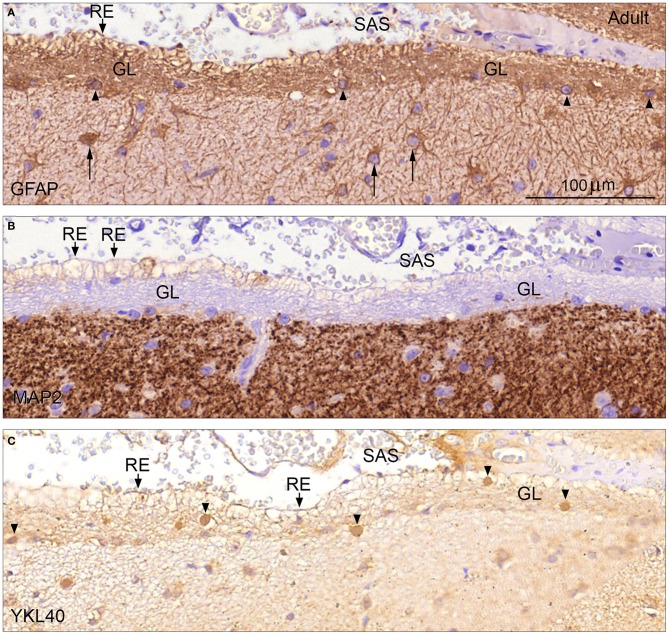
**Glia limitans in adult human brain stained for GFAP, MAP2, and YKL-40 (A–C)**. Adult human cerebral cortex immunostained for GFAP in **(A)** depicts the glia limitans (GL) as a dense multilayered network of GFAP positive astrocytic processes and few small fibrous astrocytes (*arrowheads*). Note the larger protoplasmic astrocytes (*arrows*) deeper in the molecular layer. Protrusions of astrocytic processes, which look like remnants of end feet (RE) are seen as patches facing the subarachnoid space (SAS). Immunostaining for MAP2 **(B)** defines the inner border of glia limitans (GL) toward the rest of the molecular layer and the unstained outer border with the empty-looking protrusions/remnants of end feet (RE) toward the subarachnoid space (SAS) is clearly depicted. Immunoreactivity for YKL-40 of glia limitans (GL) is seen in **(C)**. Many protrusions exhibit distinct apical membrane reactivity for YKL-40, and YKL-40 positive spheroid bodies corresponding to corpora amylacea are seen within the glia limitans but also in the border zone toward the MAP2 positive part of the molecular layer (arrowheads). (**A–C)** same magnification. **(A)** *Scale bar:* **(A)** 20 μm.

Glia limitans, which forms the outermost part of the molecular layer in adult human cerebral cortex, consisted of a dense, 20–40 μm thick network of intermingled GFAP-positive astrocytic processes and some small fibrous astrocytes, which were not surface-associated (Figure 5A). These astrocytes and the outer surface of the glia limitans were unstained following immunohistochemistry with an antibody against the neuronal marker MAP2. The positive immunoreaction clearly defined the border zone toward the rest of the molecular layer (Figure 5B). In some regions the outermost layer of the glia limitans consisted of GFAP-negative empty-looking processes reminiscent of second trimester end feet, referred to as remnants of end feet (Figures 5A–C) that protruded into the subarachnoid space. Many of these protrusions showed distinct apical membrane reactivity for YKL-40 (Figure 5C). YKL-40 staining also revealed many YKL-40 positive spheroid bodies corresponding to corpora amylacea particularly within the glia limitans but also in the border zone toward the MAP2 positive part of the molecular layer (Figure 5C) and in large perivascular spaces (not shown).

## Discussion

### The arachnoid barrier cell layer

Until recently the brain barriers in the developing brain were considered absent or leaky by many investigators (see Discussion in Saunders et al., 2014). Evidence is now emerging that these barriers are functioning and adapted to fetal environment already in the earliest stages of brain development (Saunders et al., 2012, 2013).

When the subarachnoid space is formed (see Table 2), the arachnoid barrier cells facing the inner meningeal dural border cells have closely apposed cell membranes and are joined to each other by numerous tight junctions. It has been shown in many publications that this barrier cell layer is the structural basis for the arachnoid blood-CSF barrier (see e.g., Nabeshima et al., 1975). However, so far it is not known which tight junctional proteins contribute to the paracellular diffusion restriction. We tested the arachnoid blood-CSF barrier for the presence of a number of different claudins using immunostaining and found that claudin-11 may serve as a reliable marker for the arachnoid barrier cells, as suggested from parallel finding in developing mouse brain (Whish et al., 2015). Claudin-11, initially introduced as oligodendrocyte-specific protein (OSP), is involved in formation of tight junctions and is best known for its implication in interlamellar tight junction strands in oligodendrocytes (myelin sheath tight junctions) as well as tight junction strands of the Sertoli cells of the testes (the blood-testis barrier, Morita et al., 1999; Morrow et al., 2010). Our results suggest that the arachnoid blood-CSF barrier is present and characterized by claudin-11 in early fetal life.

Since the arachnoid blood-CSF barrier is avascular and provides a relatively small surface area, it is believed that it does not contribute significantly to blood-brain exchange (Abbott et al., 2010). However, in light of recent findings of drug transporters and drug-metabolizing enzymes in the arachnoid blood-CSF barrier (Yasuda et al., 2013) and taken into account that the arachnoid barrier cell layer constitute a much larger fraction of the brain surface area during early development, it is clear that the arachnoid blood-CSF barrier is an important and changing interface, that should not be ignored. It clearly warrants further investigation in the developing and adult brain.

### YKL-40/SSEA-4 in outer CSF-brain barrier

After the penetration of the first vessels into the telencephalic wall and the appearance of the choroid plexus the following mesenchymal/neuroectodermal interfaces comprise the initial brain barrier system: (i) The blood-brain barrier proper across the cerebral endothelial cells where YKL-40/SSEA-4 are present in pericytes (Bjørnbak et al., 2014), (ii) the blood-CSF barrier at the choroid plexus epithelium that is strongly YKL-40/SSEA-4 positive (Barraud et al., 2007; Bjørnbak et al., 2014), (iii) the claudin-11 positive arachnoid blood-CSF barrier delineating the subarachnoidal space which contains strongly YKL-40/SSEA-4 positive leptomeningeal cells and vessels, (iv) YKL-40/SSEA-4 positive pial microvessel blood-CSF barrier, and (v) an outer CSF-brain barrier with a collagen 1 positive pial surface layer covering a glial end feet layer comprising BLBP/SSEA-4/YKL-40/IL-13Rα2 positive radial glial end feet. In addition, there is (vi) an inner CSF-brain barrier interface between the ventricular CSF and the brain interstitial fluid at the apical neuroepithelial cell membranes linked by strap junctions (Møllgård et al., 1987) and lined by dense YKL-40 immunoreactivity in the early developing human.

In parallel to developmental processes at the inner CSF-brain barrier, where strap junctions disappear when radial glial cells differentiate to ependymal cells (see Whish et al., 2015), the outer CSF-brain barrier seems to change character when the most superficial subpial glial end feet layer transform into a subpial glia limitans between 25 and 28 wpc in the human brain (Table 2).

An essential morphological feature of the blood-brain barrier proper (BBB) lies in the presence of tight junctions between the cerebral endothelial cells of the vasculature of the brain parenchyma. Solute transport mechanisms and restrictions have been studied thoroughly in developing and adult blood-brain barrier and choroid plexuses blood-CSF barrier, discovering distinct, comprehensive and changing transport systems, which indicate that the choroid plexuses blood-CSF and blood-brain barriers are functionally mature with respect to small molecules in early development (Saunders et al., 2013). In contrast, little is known about the developing outer CSF-brain barrier that separates the outer CSF from the brain's internal milieu. In the rat brain Balslev et al. (1997) described an incomplete layer of radial glial end feet as a part of the brain surface and large fenestrated sinusoid vessels in the subarachnoid space at E12, but there was no albumin reactivity inside the marginal zone suggesting that there must be some diffusional restriction to entry of proteins even in early development. From E14 the pia-arachnoid vessels became non-fenestrated and the developing end feet layer was connected by junctional structures that changed over time and offered restriction to protein penetration, most likely supplemented by the basement membrane (Balslev et al., 1997). In the present study we found immunostaining of the glial end feet layer with both EAAT1 (glutamate transporter), IL-13Rα2 (YKL-40 receptor) and BLBP, which transports fatty acids (Kipp et al., 2011) indicating that this outer CSF-brain barrier may well be involved initially in controlling the transfer and diffusion of solutes including morphogens into the brain and cellular transmigration (see below).

Furthermore we observed that the closely packed layer of leptomeningeal cells in the initial pial surface layer covering the radial glial end feet became less coherent in parallel with the formation of the glia limitans, indicating that the pial surface layer contributes to diffusion restriction in early development, a finding consistent with the results of Balslev et al. (1997).

*YKL-40* mRNA expression was strong in the pia-arachnoid tissue and prominent YKL-40 staining was evident already from the emergence of the meningeal tissues (Bjørnbak et al., 2014). Our results suggest that YKL-40 is produced by the leptomeninges and secreted into the subarachnoid space. Since the junctional structures between glial end feet restrict diffusion and based on the presence of the YKL-40 receptor IL-13Rα2, YKL-40 itself, as shown by the pronounced immunoreactivity of the entire end feet layer, is likely to be due to receptor-mediated uptake from the subarachnoid space.

### Involvement of arachnoid blood-CSF and outer CSF-brain barriers in brain inflammation

Until recently the central nervous system has been considered an immune-privileged site, but it is now evident that monocyte-derived macrophages also play an important role in its inflammation and maintenance of the functional plasticity of the healthy brain (Schwartz et al., 2013). The brain is under constant immune surveillance by both blood-born immune cells in leptomeningeal and perivascular spaces and resident microglia (Engelhardt and Coisne, 2011; Stolp et al., 2013). The brain barriers provide relative specialized immune privilege controlling leukocyte trafficking, which is increased considerably during inflammation and disease (Muldoon et al., 2013). Muldoon and colleagues describe four routes for leukocyte CNS entry: (1) blood-to-subarachnoid space via leptomeningeal vessels (pia microvessel blood-CSF barrier); (2) blood-to-parenchymal perivascular space through the BBB; (3) blood-to-cerebrospinal fluid via the choroid plexus (choroid plexuses blood-CSF barrier); (4) blood-to-CSF via meningeal spaces (arachnoid blood-CSF barrier) and through the ependymal lining ventricles (inner CSF-brain barrier) (Ransohoff et al., 2003; Engelhardt and Coisne, 2011; Muldoon et al., 2013; Schwartz et al., 2013). In the subarachnoid space specific antigen triggering enables the immune cell to penetrate the glia limitans (outer CSF-brain barrier) (Bartholomaus et al., 2009; Engelhardt and Coisne, 2011), which requires the activity of matrix metalloproteinases MMP-2 and MMP-9 (Agrawal et al., 2006). Thus, leucocyte trafficking involves all interfaces of the brain barrier system.

It is generally believed that inflammation produces a breakdown of blood-brain barrier at some stages of brain development (Stolp et al., 2005). Ichikawa and Itoh (2011) found that experimental meningitis in rats at first caused increased permeability of the arachnoid blood-CSF barrier rather than the BBB or choroid plexuses blood-CSF barrier indicating, that breakdown of the BBB and choroid plexuses blood-CSF barrier is secondary. However, further studies are necessary to clarify the role of arachnoid blood-CSF and outer CSF-brain barriers in response to inflammation in the developing brain. Nevertheless, the presence of an early claudin-11 positive arachnoid blood-CSF barrier, diffusion restraint through the outer CSF-brain barrier and distinct transport systems and markers found in critical barrier sites in the present study may be important for securing homeostasis and normal immune function.

### Brain barriers, inflammation and YKL-40

In a recent study of YKL-40 in the developing human forebrain, we showed a marked YKL-40 immunoreactivity in neuroepithelial cells, radial glial end feet, leptomeninges, and choroid plexus epithelial cells in early stages. Later developmental features included an increasing number of YKL-40 immunopositive pericytes particularly in the intermediate and subventricular zones and a population of small rounded YKL-40 positive possible astroglial progenitors migrating along radial glial fibers in the subventricular zone. The choroid plexus, glial end feet and the astrocyte-resembling cells observed in the developing hippocampus were strongly YKL-40 immunopositive (Bjørnbak et al., 2014). In the present study we confirmed the YKL-40 reactivity and found overlapping immunostaining of SSEA-4 and YKL-40 in leptomeningeal cells and the end feet layer.

Current knowledge suggests that neuroinflammation is of great importance in almost all neurological disorders, and that interaction between the brain barriers and the immune system contribute to this process (Stolp et al., 2013). YKL-40 immunoreactivity is specifically detected at sites of brain barriers and entry points for microglia in the developing human brain and is known to be involved in angiogenesis, inflammation and is overexpressed in glioblastoma (brain cancer, GBM) (Lal et al., 1999; Nigro et al., 2005; Colin et al., 2006) and brain tissue and/or CSF in several neurological disorders that commence in the developing brain or/and involve dysfunction of brain barriers (Chung et al., 2003; Colton et al., 2006; Arion et al., 2007; Garbett et al., 2008; Bonneh-Barkay et al., 2010). YKL-40 has also been found to regulate MMP-2 (Ku et al., 2011) and MMP-9 in some cases (Michelsen et al., 2010). The general spatio-temporal distribution of YKL-40 immunoreactivity suggests that YKL-40 secretion from choroid plexus epithelium, leptomeningeal cells and pericytes is involved in controlling local angiogenesis, access of peripheral cells (microglia and perhaps macrophages) to the developing brain and its specialized immunity. The correlation between YKL-40 and MMPs, presence of YKL-40 in brain barriers and necessity of MMP-2 and MMP-9 in immune cell transmigration through the glia limitans further support the hypothesis, that YKL-40 has a role in immune cell transmigration across brain barriers. Whether YKL-40 expression in the brain barriers is increased in response to neuroinflammation warrants further investigation.

### Stem cells, brain barriers and SSEA-4

In a previous study of human embryonic central nervous system, the stage-specific embryonic antigen-4 (SSEA-4) was found in neural progenitor cells in forebrains of human embryos and early fetuses (Barraud et al., 2007), and was shown in a population of differentiating cells in an intermediate stage between pluripotent human embryonic stem cells (hESCs) and neural progenitor cells (Noisa et al., 2012). SSEA-4 belongs to the group of globo-series glycosphingolipids, and is widely used in characterization of hESC lines (Adewumi et al., 2007). Furthermore, it is highly expressed in GBM compared to normal tissue and an SSEA-4 specific antibody suppresses GBM tumor growth in mice (Lou et al., 2014). In other cancers SSEA-4 has been established as a cancer stem cell marker (Malecki et al., 2012; Noto et al., 2013). The glycoprotein YKL-40 has been associated with hESC and neural stem cell differentiation (Brøchner et al., 2012), and a marked similarity of the distribution patterns in developing human forebrain was noted (Barraud et al., 2007; Bjørnbak et al., 2014). In this study the localization of YKL-40 and SSEA-4 was almost identical corresponding to earlier findings. The role of SSEA-4 in brain barriers remains to be elucidated but its known presence in hESC, GBM and cancer stem cells and co-localization with YKL-40 in the brain barriers suggest that the outer brain barriers participate in organization and development of the brain through several different mediators including SSEA-4.

### BLBP and the morphogenetic role of meninges

Brain specific lipid-binding protein (BLBP) is a member of the lipid-binding protein family that carries small hydrophobic signaling molecules between cellular compartments but also a marker of radial glial cells (Feng et al., 1994; Howard et al., 2006), astrocytes (Kipp et al., 2011) and adult neural stem cells (mice) (Giachino et al., 2014). BLBP expression is increased in brain tumor tissue and associated with progression and/or decreased survival in glioblastoma (GBM). Depending on the ratio of fatty acid ligands BLBP can increase or decrease migration in GBM (Kipp et al., 2011). A variety of secreted diffusible factors from fetal meninges regulate neural migration and positioning and organize the pial basement membrane, which is a critical anchor point for the radially oriented fibers of neuroepithelial stem cells (Siegenthaler and Pleasure, 2011). Signaling molecules identified so far include the chemokine SDF-1, BMP7, and the morphogen all-trans retinoic acid (atRA) which seems to be involved in the decision of neuroepithelial cells to generate neurons and intermediate progenitor cells (IPCs) destined for the subventricular zone via communications with adjacent radial glial end feet (Siegenthaler et al., 2009). Feng et al. (1994) suggested that BLBP mediates aspects of neuron-glia interaction and is involved in neural differentiation and maintenance of the glial fiber scaffold. We found BLBP in the end feet layer in the developing outer CSF-brain barrier, establishing BLBP as a reliable marker for the radial glial end feet layer and thereby the outer CSF-brain barrier. Furthermore, BLBP has a preference for binding long chain polyunsaturated fatty acids with a possible ligand (Xu et al., 1996) being docosahexaenoic acid, also involved in CNS development (Xu et al., 1996), which in light of our findings support that BLBP in the outer CSF-brain barrier participate in neural development and positioning possibly also orchestrated from the meninges.

## Concluding remarks

Research regarding the brain barrier system has evolved considerably over the last decades and now the focus is on both physical, transport, and metabolic barrier properties. In this study we described the changing barriers at the surface of the developing brain, but to characterize and understand these complex structures and their interaction, we found it necessary to revise the barrier concept based merely on anatomical features. Furthermore, the terminology in this area is at best confusing and inconsistent. Based on these considerations we argued that the outer brain barriers consist of (i) an arachnoid blood-CSF barrier across the arachnoid barrier cell layer, (ii) a pial microvessel blood-CSF barrier across the pial microvessels and (iii) an outer CSF-brain barrier across the radial glial end feet layer covered by a pial surface layer and later in development glia limitans with accompanying pial basement membrane. Our findings of claudin-11 in the arachnoid blood-CSF barrier and EAAT1 and IL-13Rα2 in the outer CSF-brain barrier confirm their barrier properties and the spatiotemporal distribution of YKL-40/BLBP/SSEA-4 further establish the necessity of regarding these interfaces as separate entities, since they may play different roles in normal brain development and possibly in several neurological disorders. We therefore propose an elaboration of the model for developing brain barriers as defined by Saunders et al. (2008) and divide the brain interfaces into six barriers at three different sites: (1) The blood-brain barrier proper (i), (2) the blood-CSF barrier constituting (ii) the choroid plexuses blood-CSF barrier across the choroid plexus, (iii) the arachnoid blood-CSF barrier across the arachnoid barrier cell layer and (iv) the pial microvessel blood-CSF barrier across the pial microvessels; (3) The CSF-brain barrier comprising (v) the outer CSF-brain barrier (described earlier in this paragraph) and (vi) inner CSF-brain barrier across the ventricular zone cell layer.

### Conflict of interest statement

The Review Editor Gavin Clowry declares that, despite having collaborated with author Kjeld Mollgard, the review process was handled objectively. The authors declare that the research was conducted in the absence of any commercial or financial relationships that could be construed as a potential conflict of interest.

## Abbreviations

BBB: blood-brain barrier
BLBP: brain specific lipid-binding protein
CSF: cerebrospinal fluid
EFL: end feet layer
EM: electron microscopy
GFAP: glial fibrillary acidic protein
PSL: pial surface layer
SAS: subarachnoid space
SSEA-4: stage-specific embryonic antigen-4.
